# Behaviourally specialized foragers are less efficient and live shorter lives than generalists in wasp colonies

**DOI:** 10.1038/s41598-019-41791-0

**Published:** 2019-03-29

**Authors:** Davide Santoro, Stephen Hartley, Philip J. Lester

**Affiliations:** Victoria University of Wellington, School of Biological Sciences, Wellington, New Zealand

## Abstract

A widely held assumption in ecology is that specialists are more efficient than generalists. However, empirical evidence for this fundamental assumption is surprisingly scarce and often contradictory. Theoretically, the evolution of alternative life history strategies is underpinned by a trade-off between activity levels and survival. We investigated the consequences of specialization in a foraging context, by comparing the performance and longevity of closely related individuals in a social insect, the common wasp (*Vespula vulgaris*). Using radio-frequency identification technology, we monitored the lifetime foraging activity of individual wasps from three colonies kept under natural foraging conditions. Returning foragers were video-recorded as they passed the nest entrance so that their foraging load could be assessed. There were substantial differences in foraging activity and survival within and between colonies. At the colony level, foraging specialization was weak. Yet, workers within each nest demonstrated a remarkable range of foraging specialization levels (defined as the degree of overlap between individual and colony-level task allocation) and efficiencies (defined by the number of successful trips and trip duration). We found that specialist foragers were less efficient than generalist siblings within the same colony. Behavioural specialists accomplished fewer successful trips per foraging day, and their trips were typically relatively longer. Specialized foragers also showed reduced life expectancy. The mortality risk was higher for individuals spending relatively more time in the field, yet we found no link between the level of specialization and relative field exposure. Our extensive dataset of unprecedented detail provides strong empirical evidence that behavioural specialization is not associated with a better lifetime performance, on the contrary, the opposite appears true for the common wasp. We also show that the survival of genetically similar individuals can be linked to life-long differences in behaviour according to classical life-history theory predictions.

## Introduction

A fundamental assumption in ecological and evolutionary studies is that specialists are more efficient than generalists^1^. This critical assumption implies that specialists have evolved physiological, morphological or behavioural traits that lead to a relatively greater efficiency in specific resource exploitation^2–4^. Specialization can be defined according to different conceptual frameworks and investigated at different levels^3^. Some empirical evidence exists from both interspecific^4–6^ and intraspecific studies^7,8^ that specialists have evolved behavioural adaptations to handle their preferred food types more efficiently. Yet, the investigation of the consequences of ecological specialization is still very limited, especially amongst genetically-related individuals^9^.

Insect societies represent unique study systems, as individuals within colonies are highly related, and selection operates both at the individual and at the colony level^10^. Social bees, social wasps, ants and termites may make up an estimated 75% of the world’s insect biomass^11^. The division of labour among workers is considered to be a major reason behind the ecological success of eusocial insects (characterized by reproductive division of labour, overlapping generations, and cooperative brood care)^12,13^. Theoretically, to develop, evolve, and to be maintained, the differential task allocation among individuals and their consequent specialization are expected to increase relative colony fitness. Two mutually non-exclusive mechanisms for increased efficiency at the colony level are via group task partitioning advantages, or via increased individual efficiency in task performance^14^. Social insect workers can switch tasks according to their age (temporal or age polyethism), and these changes (polyethic transitions) can be more (e.g. honey bees) or less (e.g. vespid wasps) abrupt^15,16^. Hence, to show that specialization exists, it is not sufficient to quantify how often an individual performs a single task. Instead, it is necessary to show that workers preferentially perform one task compared with other nestmates^11^. It is also fundamental to focus on the consistency of individual behaviours^13,17^, and to consider the different time scales across which specialization can occur^11^.

Insect workers specializing in a particular task are commonly assumed to be more efficient than those frequently switching tasks in response to the changing colony needs^11,13^. Considering multilevel selection, though, social insect foraging does not necessarily fit into “optimal foraging” models, developed for animals that optimize their resource-collecting activity in accordance with their individual needs^18^. For example, foraging workers could sacrifice their individual performance in exchange for a faster return to the colony and the activation of additional foragers^18,19^. Very few empirical studies have investigated the efficiency of individual workers and how individual efficiency relates to their task specialization^11,20,21^. Both in ant and bumble bee colonies, workers’ specialization in particular tasks was unrelated to their ability to perform them^11,21^. Yet, there is increasing evidence of enormous and consistent differences in activity amongst insect nestmates^22–25^. Some individuals, referred to as “elite workers”, are extremely active and productive^22,23,25^, while others are comparatively “lazy”^26^.

A major determinant of animals’ fitness is their foraging behaviour. For insect colonies, foraging is a fundamental requirement in order to grow (by producing workers) and to reproduce (by producing reproductive individuals). For the individual worker, foraging is a risky and costly activity^23,24^. The age at first foraging (foraging onset) is known as a central variable in in a social insect worker’s life history^24,27^. For example, precocious honey bee foragers show higher risk of death in their first flights^23^. Independently from their age at first foraging, wasp workers from *Polybia* colonies lived an average of six days after foraging onset, and the length of their foraging career did not decrease as age of first foraging increased^24^. Consistent with a trade-off between foraging activity and lifespan, foraging seems to be undertaken by individuals with reduced life expectancy and residual value^28^, e.g. older honey bees^23^, or wasps in subordinate social positions^29^.

The high mortality of foragers can be explained by a combination of extrinsic and intrinsic factors^27^. Foraging individuals leave a relatively safe nest and expose themselves to an unpredictable and risky environment, where death can be caused by external hazards such as predation, accidents, dehydration, or disorientation^27^. The accelerated senescence and premature death of foragers can derive from wear-and-tear caused by foraging bouts^28^. Foraging is also energetically expensive, and particularly costly when it involves powered flight^30,31^. Hence, individual foraging is thought to enhance colony fitness at the expense of a forager’s relative intra-colony fitness^29^.

Here, we focus on the consequences of foraging task specialization on individual life history. We examine how behavioural specialization in foraging activities can predict individual efficiency and longevity, taking into account potential confounding factors (colony, individual size, and foraging onset). We studied the common wasp *Vespula vulgaris* (Linnaeus, 1758). Foragers collect resources including solid protein for larval rearing, liquid carbohydrates for energy and thermoregulation, and wood or ‘pulp’ for nest building^32^. By combining longitudinal observational data and a lifetime of continuous records obtained through automated monitoring, we quantified the degree of foraging task specialization within colonies. We addressed two main questions in this study. Firstly, are specialist foragers more efficient than generalists, making more frequent and shorter foraging trips? Secondly, how is the level of foraging specialization linked to individual life expectancy and time spent in the field, and does such field exposure increase mortality risk?

## Methods

### Study organism and experimental set up

The common wasp *V. vulgaris* (Hymenoptera: Vespidae) is an eusocial insect species native to Eurasia, and invasive to New Zealand and other Southern hemisphere countries^33^. Workers are monomorphic but can vary in size within the same colony^34^. Worker-worker relatedness is variable, as queens can mate with multiple males. Such polyandry is considered rare among social Hymenoptera^35^. There is some evidence that workers of *Vespula* and *Dolichovespula* spp. (“yellowjackets”) can specialize in a particular foraging task on different time scales^15^.

Our study was conducted on three *V. vulgaris* colonies, during 2014 and 2017, in New Zealand (see SI and Table S1 for details). When collected, these colonies were in a rapid expansion phase. Each colony had one queen and a thousand or more workers, foraging to expand the nest and get to the production of males and new queens. On the day of collection, the nests were transported to the research facilities. The colonies were then immediately anesthetized with carbon dioxide and relocated with the nests into wooden boxes. The wasps could freely forage in the field passing through a transparent acrylic glass entrance attached to the nest box (Figs S1–S3). This entrance module was 60 cm long and shaped as a double funnel to direct the traffic into two separate lanes, one for the wasps going out, and one for those returning from foraging trips (“incomers”). The module contained one radio-frequency identification (RFID) system (ilD®HOSTtypeMAYA4.1, microsensys GmbH). Two RFID tunnel scanners (ilD®MAYAreadermodule4) were placed in series in each lane, in order to identify individuals provided with RFID tags, their direction of travel, the time of their entrance or exit, and minimize the possibility of missing records. RFID traffic data were obtained continuously over the entire study period.

From each colony, two combs with pupae were kept aside in an incubator (30 °C and complete darkness) as a source of known-age workers. All the wasp adults emerging from the incubated combs had either RFID tags (mic3-TAG 16 kbit, microsensys GmbH) or (for 330 individuals in 2014) numbered tags (Queen Numbering Kit, Ecroyd Beekeeping NZ) glued to their backs with a ethyl cyanoacrylate based adhesive (Quick Fix Gel Supa Glue, Selleys). To estimate the body size of these tagged individuals, we measured their head width, using a digital calliper to the nearest 0.01 mm. Overall, 1657 tagged and measured individuals were introduced into their colony of origin within 24 hours of emergence (Table S1).

The incomers’ lane of the nest entrance modules was continuously filmed (Figs S1, S2). We observed and examined a selection of videos covering every day of each colony’s activity, for a total of 465 focal hours (2014: daily, one to five hours, 8 am–8 pm; 2017: continuous, 8–9 am, 12–3 pm, 6–7 pm). During video analysis, whenever a tagged wasp passed by the first of the two readers in the incomers’ lane, the load carried and time to the nearest second were recorded (Table S1). These observational load records were subsequently matched with continuous, automated RFID traffic data, and each resource item was attributed to an individual based on the time-stamp. We also recorded the loads of all incomers (tagged and not) during ten-minute intervals for each focal hour.

The study colonies grew and thrived during the experiments. Colony A (2014) was studied for 57 days, while colonies B and C (2017) were studied for 39 and 29 days, respectively (Table S1). At the end of the study, we assessed the growth of each nest. The overall number of nest cells increased between two- to four-fold from the beginning of the study, and all three colonies got to the construction of queen cells. In 2017, the nest boxes containing colony B and C were closed at night, and frozen at −80 °C within one hour. The nest entrance modules were kept in place for the following week, to trap and scan wasps returning to the nest after its removal (Fig. S2). The two nests were later pulled apart, and the wasps examined one by one. All the tagged individuals recovered in the nests and entrance modules were identified.

### Division of labour and individual foraging task specialization indices

From the video recordings, we were able to quantify the proportion of trips made to collect one of three possible resource types: fluid, pulp or flesh. These proportions were calculated at the individual-level and at the colony-level.

To quantify the degree of division of labour (DOL) at the colony level, we used the equations and calculation tools provided by Gorelick *et al*.^36^. The DOL indices are derived from Shannon’s mutual entropy/information theory and allow to determine (i) the degree to which individuals specialize on only one or a subset of the tasks available (individual predicts task: DOL_*i→t*_), (ii) the degree to which certain tasks are *consistently* performed by a particular subset of individuals (task predicts individual: DOL_*t→i*_), and (iii) the overall division of labour (DOL_*Total*_). DOL index values range from 0 to 1. The higher the value, the stronger the degree of division of labour. For the DOL indices, we included all tagged foragers observed with at least five loads, to make our results directly comparable to a previous review^37^.

To quantify the degree of individual task specialization of each foraging wasp, we used the Petraitis’ (1979) index W_*i*_, calculated using the program *Indspec1*^38^. W_*i*_ measures the degree of niche overlap between each individual and the population average^38^. W_*i*_ values range from 0 to 1. The lower the W_*i*_ value, the less overlap between a given individual’s behaviour and the colony average, hence the higher the degree of specialization. We accounted for the potentially confounding effects of polyethic transitions in task allocation by calculating W_*i*_ for each individual (*i*) considering the age (*a*, days post emergence) when the last trip was performed. We measured the degree of overlap between the life-time load spectrum for *i* and the spectrum of all the known-age nestmates including *i*, considering only the loads collected to the age *a*. We hence obtained an individual measure of colony-specific, age-sensitive foraging task specialization, quantifying the likelihood of each individual’s task being drawn from the general proportions of foraging tasks observed at the colony level (Fig. S4). For W_*i*_, we included all RFID-tagged individuals observed with at least nine loads. With this threshold, any individual observed performing only one task had W_*i*_ value below the colony median.

### Individual activity, longevity, efficiency, and field exposure measures

The RFID automated monitoring enabled us to calculate the duration of each sortie outside of the nest for all the known-age wasps provided with RFID tags. To avoid confusing effects from circling, drifting and overnighting episodes (see results), we considered “foraging trips” sorties longer than two minutes and shorter than eight hours (personal observations showed that two minutes was the minimum time necessary to walk through the entrance both ways and take a resource in front of the nest entrance, while the time window of eight hours roughly corresponded to the night-time foraging stop of the wasp colonies). For each individual, we were able to determine the length of each foraging trip (in seconds), the number of lifetime trips, and obtain an estimate of the age at first foraging (foraging onset), foraging tenure (number of foraging days), and adult lifespan (*l*, from the day of emergence to the day of the last RFID record). This longevity proxy was reasonably validated by the RFID data combined with the tagged individuals recovered in 2017 (see results).

We measured individual efficiency and foraging performance considering two proxies, the average number of trips per foraging day, and the individual average standard trip length. The *average number of trips per foraging day* (ANTFD) was preferred to the number of lifetime foraging trips because, for example, some individuals might make more trips than others by virtue of a longer life and/or foraging tenure. The duration of a foraging trip can be considered a proxy of how much is accomplished per unit time. Assuming homogeneity in load size and/or nutritional value (different currencies, building and food materials) outperforming individuals would make trips shorter than others. Hence, we also measured efficiency as a function of trip length, elaborating the *average standard trip length* (ASTL). A standardized measure was needed as trip duration can vary in time (e.g. for changing weather conditions or resource availability), and different resources can require different collecting times. Firstly, to control for daily and seasonal variation in trip length, we calculated for each individual *i* the average relative trip length (ARTL*i*), measured as the average of all the trip lengths, each one divided by the average trip length of all the active foragers at any corresponding day and hour. Secondly, to take into account the different times needed to obtain different load types, we calculated the average relative trip length (ARTL*j*) for each load type *j* (fluid, pulp, flesh), dividing the average trip length (ATL) for each load type (ATL*j*) by the lowest ATL*j* so that ARTL*j* ≥ 1. Then, to take into account the individual task allocation and its potential influence on ATL, an individual correction factor K*i* was calculated as K*i* = ΣP*ij* * ATL*j*, where P*ij* is the proportion of load type *j* for the individual *i*. Finally, the individual average standard trip length was calculated as ASTL*i* = ARTL*i*/K*i*.

We measured field exposure as the *relative field exposure (RFE)*. This standardized measure was elaborated as the time spent outside the colony can vary as a function of age. For each colony, we obtained the average time spent out in the field at any given age *a*, considering all RFID-tagged individuals alive at age *a*. We were then able to calculate the cumulative average field time at any given age (CAFT*a*). For each individual *i* with a certain lifespan *l*, we summed the duration of all the sorties out of the nest, obtaining the total time spent in the field (TFT*i*). Finally, the individual relative field exposure was calculated as RFE*i* = TFT*i*/CAFT*a*, with *a* = *l*.

### RFID data processing and statistics

Filtering and processing of RFID data were performed with *Track-a-Forager1.0* (cut-off settings: cluster = 20 s, in/out = 20 s, flight minimum = 10 s, flight maximum = unlimited time)^39^.

For the statistical analysis, we used R 3.5.2^40^. To test whether the degree of task specialization measured by W_*i*_ was linked to foraging efficiency, measured as ANTFD or ASTL, we first log-transformed (log10) the two variables, as both had an extremely skewed distribution (Shapiro-Wilk normality test W = 0.70, *p* < 0.0001; W = 0.71, *p* < 0.0001 respectively). Then, we used linear models including W_*i*_, colony, head width and foraging onset as fixed factors. We also considered each colony separately and used simple linear models to regress W_*i*_ with ANTFD and ASTL (log transformed). We performed a multivariate Cox regression survival analysis to investigate if and how the degree of foraging task specialization was linked to mortality. W_*i*_, colony, head width and foraging onset were included as covariates. To test whether the degree of field exposure measured by RFE was linked to task specialization and mortality, we first log transformed the variable, as it had an extremely skewed distribution (Shapiro-Wilk normality test W = 0.59, *p* < 0.0001). We considered each colony separately and used simple linear models to regress W_*i*_ with logRFE. We performed a univariate cox regression survival analysis to test if and how logRFE was linked to individual life expectancy.

## Results

### Wasp foraging behaviour, longevity, and division of labour

**Figure 1:**
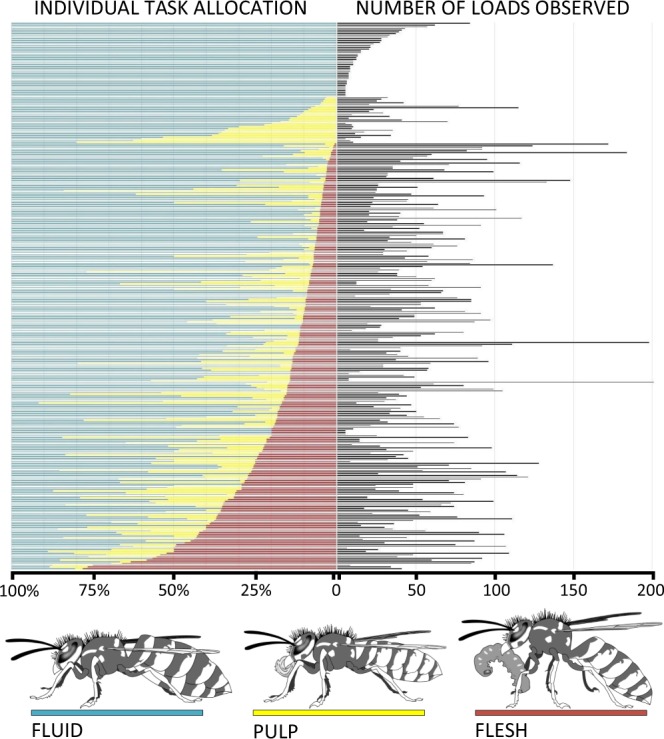
(left) Inter-individual variation in foraging task allocation among common wasp (*Vespula vulgaris*) workers (colony A). (right) Number of loads observed for each wasp throughout its life and foraging career (only individuals observed with five or more loads are represented). Each horizontal line represents one tagged wasp worker. Rows ordered first by proportion of flesh loads (minimal to maximum), then by pulp loads, and finally by number of loads observed. (bottom) drawings representing wasps with the load types distinguished (by David Young, 2016). These life-long observational data were obtained continuously video-recording the wasp nest entrance (Figs S1–S3), and used to calculate the degree of division of labour at the colony level (Table 1) and specialization at the individual level (Table 2, Figs 2, S4).

**Figure 2:**
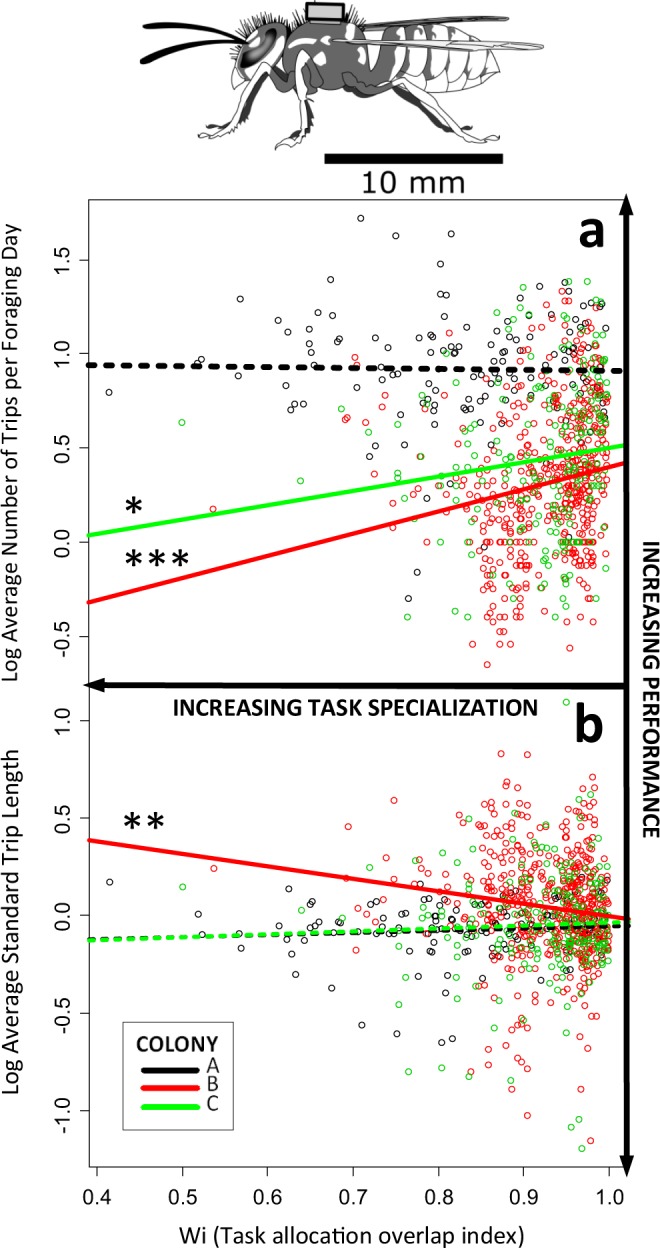
*Vespula vulgaris* individual foraging specialization in relation to performance over lifetime, measured as (**a**) *average number of trips per foraging day* (ANTFD) and (**b**) *average standard trip length* (ASTL), log transformed. These life-long data were obtained using RFID tags glued on the wasps’ thorax (top drawing - David Young, 2016). Dots represent individual measure averages, colours colony of origin, and lines regressions for each colony. Dashed lines indicate non-significant relationships, asterisks refer to significant linear relationships *P < 0.05, **P < 0.01 and ***P < 0.001. For our multifactorial model, we included W_*i*_, colony, head width and foraging onset age as fixed factors (Table 2).

Overall, we recorded the outcome of 101,800 wasp foraging trips (Table S1). In general, the prevalent foraging task at the colony level was represented by fluid foraging, with 55% of the returning foragers showing a swollen abdomen and no solid loads in their mandibles. Pulp loads accounted for 24% of all the loads. Flesh was carried by 19% of the returning foragers (Figs 1, S1, Table S1). In 2% of the cases, incomers were observed with no solid loads and empty crops (Fig. 2).

We found enormous variability in the length of the foraging trips performed by the same individual throughout its life and among different individuals at any one time. The time spent outside of the nest ranged from 10 seconds (when individuals circled in the nest entrance module, walking out of the nest box and immediately re-entering it) to 19 days. Individuals spending multiple days and nights outside the natal nest may have joined other colonies (“drifting”). In fact, four marked individuals were seen foraging for non-natal colonies in 2014, and two individuals drifted between colonies B and C in 2017, spending multiple days in both colonies. Drifting has been observed in other social wasp species^41,42^. Overall, 38% of the wasps foraging spent more than 8 hours out in the field at least once during their lifetime, most frequently overnight, and 9% of the foragers spent more than 24 consecutive hours outside of their nest (we verified on a subsample of raw RFID records that the longest sorties were not artefacts of data filtering and postprocessing)^39^.

Of the 1327 individuals provided with RFID tags and inserted into the three colonies, 13 (1%) were never recorded and could have i) spent their whole life inside the nest and died without ever walking out of the nest entrance, ii) stayed inside the nest until the end of the experiments (two individuals in 2017, see below), iii) gone out undetected and did not return, or iv) lost their tag. Foragers were observed and recorded up to their 50th day of life (18.8 ± 7.7 days), and 78% of the RFID tags were last recorded by the scanners on the way out of the nest. The last RFID record can be considered a good estimate of lifespan. In fact, the wasps studied kept their nests clear of paper waste, remains of prey, faecal material, and dead larvae and adults (the level of nest sanitation can differ between *Vespula* species^42^ and be lower in colonies with a weak workforce or close to collapse, personal observation). Dead workers were promptly carried out of the nest partially eaten or dismembered (those with RFID tags were sometimes recorded by the scanners). In 2017, we tagged 1027 wasps, and at the end of the study we terminated colonies B and C by freezing, recovering 124 (12%) of the tagged individuals. All but two of these workers were intact, and most likely alive when the colonies were frozen. Only the RFID tags of seven individuals (one missing head and thorax) had been recorded more than 24 hours before the colonies were frozen. Two additional individuals (intact, one from colony B, the other from colony C, and respectively 20 and 18 days old) had never been recorded.

We could identify and measure the length of 63,757 “foraging trips” (defined as sorties longer than two minutes and shorter than eight hours), performed by 1100 RFID-tagged foragers (17% of the wasps provided with RFID tags made no foraging trips, making no sorties or sorties shorter than two minutes). Substantial differences in foraging effort and survival were found among foragers (Fig. 2, Table S1). Wasps performed their first foraging trip between 1 and 23 days after adult emergence (mean = 7.3 ± 2.8 SD days). The foraging tenure lasted up to 43 days (9.9 ± 7.9 days). The number of lifetime trips varied enormously, and wasp workers could perform hundreds of trips during their life (57.2 ± 106.3; range = 1–805) (Fig. 2a). The foraging trip length varied widely (21 ± 30 min), and depended on the type of load, being likely influenced by resource availability in the field (Table S1, SI).

The DOL indices measured a weak degree of foraging specialization at the colony level (DOL_*Total*_ range = 0.03–0.09), and the foraging task predicted the individual rather than vice versa (DOL_*t→i*_ > DOL_*i→t*_, Table 1). Wasp workers showed foraging task distributions covering almost the entire potential spectrum. Most individuals undertook all foraging tasks without a clear preference. Some individuals, however, specialized on one foraging task (fluid foraging) throughout their entire adult life (Fig. 1). Inter-individual variability could not be explained by polyethic transitions, as the specialization index adopted considered polyethism as a confounding factor (Fig. S4). A subset of 848 foragers with RFID tags were observed with at least nine loads and were hence considered for the main analysis of specialization in relation to foraging performance and survival.

**Table 1:** Division of labour (DOL) indices for *Vespula vulgaris* foraging tasks.

Colony	N tasks	N individuals	DOL_*i→t*_	DOL_*t→i*_	DOL_*Total*_
A	3	317	0.03	0.23	0.09
B	3	567	0.01	0.09	0.03
C	3	279	0.01	0.11	0.04

### Individual foraging task specialization and efficiency: do more specialized wasps perform more trips? Are these individuals making shorter trips

We found that specialists were less efficient than generalists (Fig. 2). Our multi-factorial models revealed that more specialized wasps (with lower W_i_ values) tended to perform fewer trips per foraging day (lower ANFTD), and that the average standard trip length (ASTL) was not shorter than that of more generalist nestmates (Table 2). For each colony, we also regressed the degree of niche overlap between each individual and the population average (W_i_ index) with both ANTFD and ASTL (log transformed). We found that more generalist wasps tended to accomplish more daily trips than their specialized nestmates in colonies B (multiple R^2^ = 0.034, R^2^adj = 0.032, F_1,499_ = 17.36, p < 0.0001) and C (multiple R^2^ = 0.019, R^2^adj = 0.014, F_1,210_ = 3.99, p < 0.05), but not in A (multiple R^2^ = 0.000, R^2^adj = −0.007, F_1,134_ = 0.05, p = 0.823) (Fig. 2a). In colony B, the more specialized the individual, the longer the average foraging trip relative to the others (multiple R^2^ = 0.019, R^2^adj = 0.017, F_1,499_ = 9.39, p < 0.01) but not in A (multiple R^2^ = 0.009, R^2^adj = 0.001, F_1,134_ = 17.36, p = 0.276) and C (multiple R^2^ = 0.002, R^2^adj = −0.003, F_1,210_ = 0.32, p = 0.574) (Fig. 2b). In summary, we found no evidence for the hypothesis that specialist foragers were more efficient than generalists in their foraging behaviour. If anything, generalists were more efficient, making shorter and more frequent foraging trips, but not in every colony.

**Table 2:** Multifactorial regression analysis investigating the relationship between the degree of individual foraging task specialization (measured as W_*i*_, lower values representing higher specialization) and lifetime performance (i. *average number of trips per foraging day* (ANTFD) and ii. *average standard trip length* (ASTL), log transformed). We included colony, foraging onset, and size (measured as head width) as additional factors.

Dependent variable	Predictor	Estimate	SE	*t*	
**Average Number of Trips per Foraging Day**	Individual specialization (W_*i*_)	0.545	0.169	3.220	**
	Colony (A vs B)	−0.638	0.039	−16.183	***
(LogANTFD)	Colony (A vs C)	−0.487	0.044	−10.962	***
**R** ^**2**^ **adj = 0.265**	Head width	0.676	0.206	3.291	**
	Foraging onset	−0.006	0.005	−1.334	ns
**Average Standard Trip Length**	Individual specialization (W_*i*_)	−0.134	0.119	−1.122	ns
	Colony (A vs B)	0.116	0.028	4.156	***
(LogASTL)	Colony (A vs C)	0.013	0.031	0.409	ns
**R** ^**2**^	Head width	−0.494	0.145	−3.413	ns
	Foraging onset	0.003	0.003	0.772	***

### Patterns of mortality: how is the level of foraging specialization linked to individual life expectancy and field exposure? Does the risk of mortality increase with increased field exposure

**Figure 3:**
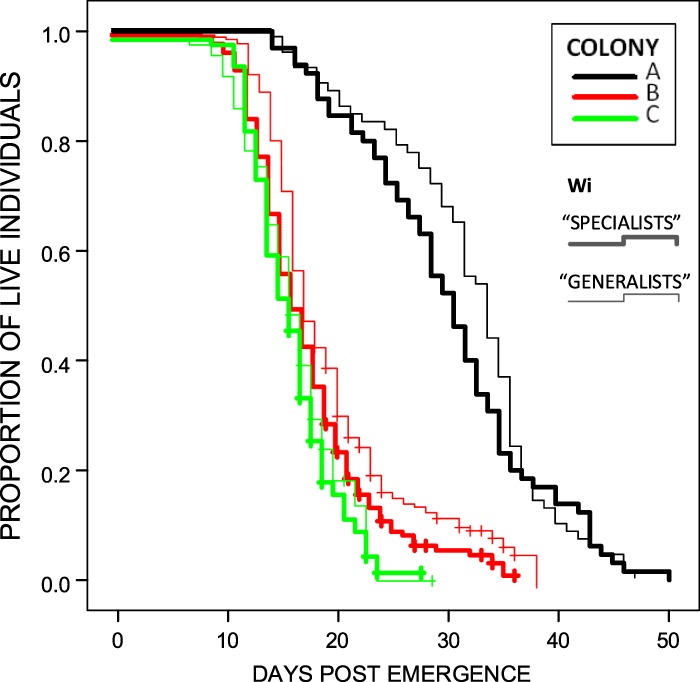
Survival curves Colonies A, B, C. Thick lines represent “specialists”, individuals grouped by W_*i*_ index, with values below the colony median. Individuals with W_*i*_ values above the colony median were “generalists”. Colours represent different colonies. Crosses refer to censored individuals from colonies B and C, recovered at the end of the data collection. For our multifactorial model, revealing that more specialized individuals lived shorter lives, we performed Cox regression survival analysis, including W_*i*_ as a continuous variable and colony, head width, and foraging onset as covariates (Table 3).

We found that more specialized foragers had a significantly shorter life expectancy (Fig. 3, Table 3). On average, “specialists” (individuals with W_i_ values below the colony median) had a 6% shorter lifespan compared to “generalists” (individuals with W_i_ values above the colony median). Larger foragers (head width measures above the colony median) experienced lower mortality risk, having a 5% longer lifespan compared to smaller individuals (head width measures equal or below the colony median). At the extremes of the size continuum, the largest workers lived on average 24 days, while the smallest 14 days. Worker survival appeared to benefit by extending the amount of time between emergence from the pupal stage and the first foraging trip. In fact, individuals who were younger at first foraging had shorter life expectancy (Table 3).

**Table 3:** Cox survival analysis investigating the relationship between the degree of individual foraging task specialization (measured as W_*i*_, lower values representing higher specialization) and lifespan, including colony, foraging onset, and size (measured as head width) as additional factors.

Dependent variable	Predictor	Coef	Hazard ratio	SE	z	
**Lifespan**	Individual specialization (W_*i*_)	−1.954	0.142	0.467	−4.186	***
	Colony (A vs B)	1.530	4.620	0.129	11.890	***
	Colony (A vs C)	1.802	6.062	0.148	12.143	***
	Head width	−1.589	0.204	0.609	−2.610	**
	Foraging onset	−0.052	0.950	0.013	−3.833	***

We found no link between the level of specialization and the relative field exposure (colony A: multiple R^2^ = 0.008, R^2^adj = 0.000, F_1,134_ = 1.02, p = 0.3144; colony B: multiple R^2^ = 0.000, R^2^adj = −0.002, F_1,499_ = 0.09, p = 0.7613; colony C: multiple R^2^ = 0.000, R^2^adj = −0.005, F_1,210_ = 0.05, p = 0.827). Wasp mortality risk increased with relatively higher levels of field exposure (higher RFE) (coef: 0.309; Hazard ratio (Exp(B)): 1.363; Wald statistics: 10.5, df = 1, p = 0.001). There were, however, substantial differences in survival between colonies (Table 3, Fig. 3). In colony A, the average lifespan of foraging wasps was 31 days. Compared to this colony, life expectancy in colony B and C was respectively reduced by 45% and 52% (but see Table S1).

## Discussion

Overall, common wasp colonies showed a weak degree of foraging specialization. Most workers within a colony opted for generalist task repertoires over their entire life, yet some foragers showed a high degree of task specialization. In fact, some individuals were observed foraging exclusively for fluids, while others collected disproportionate amounts of pulp or flesh throughout their life. Although foraging task specialization (“fixation”) has been reported on variable time scales in various eusocial insects^17,21,43^, lifelong specialization on a single task is considered rare among social insects^11,44^. We found no empirical evidence that behavioural specialization was associated with more efficient foraging performance of individual wasp workers. These results support the evolutionary maintenance of colonies composed of workers with varying degrees of specialization: if being a specialist in a colony was always beneficial, over evolutionary time we would expect these wasps to have developed colonies with distinct classes of specialized foragers.

Task specialists are frequently thought to be more efficient^11,13^. Increased efficiency in specialization may be due to a minimization of switching costs^13^, or to cognitive constraints^7,8^. Switching tasks according to the colony needs requires time to gather information within the social context^21^, spatially move, or acquire appropriate dexterity. Indeed, success in a foraging activity such as hunting can be difficult, also for a generalist and well-armed predator such as a yellowjacket^12,34^. Wasp foragers rely on knowledge and skills that must be learned^42,45^. Despite the hypothesized benefits associated to specialization, we show that when compared to specialists, generalist wasp foragers can accomplish more foraging trips, that their trips can be shorter, and their lives longer.

From the individual point of view, the preferable option minimizes personal risks and energy expenditure and maximises personal benefits. Not all individuals have the same information and the same number of options. For example, different workers within a colony would likely have a diverse knowledge of profitable resource patches, or safe foraging routes. Foraging hymenopterans are known to return to rewarding locations and show different motivation to forage for particular resources^46,47^. Hence, individuals might leave the colony aiming to collect a specific resource. But if collecting that resource became too risky, time or energy consuming, workers might opportunistically decide to gain an alternative and still valuable currency for the nest. In yellowjackets, foraging tasks are partitioned, and food items can be exchanged for concentrated nutrients, obtained both from adults and larvae^29,32^. The decision to switch to an alternative task might be delayed in individuals highly motivated to gain a specific resource in the field. These specialists could be expanding their foraging range, spend more time searching for a given resource, and pay a price in terms of overall performance and survival.

We can only speculate on the exact mechanisms causing individuals to adopt generalist or specialist life histories. Within insect colonies, the mechanisms causing differential task allocation can operate at multiple levels, and be genetic, maturational, nutritional, or of environmental nature^13,44^. Also in insects, learning processes, long term memories, and deliberate choices seem to play a very important role throughout life^13,44,46,47^. We do know that individual experience alone can shape behavioural differentiation in ants^48^ or the individual performance of wasps^45^. Greater efficiency associated with behavioural generalism could result from individual economic choices in an ever-changing environmental context^44^. Theoretically, while a relatively static environment would favour specialization, a dynamic one would favour generalism^1^. Generalism would be also promoted when switching costs are low^10^.

Specialist foragers lived shorter lives than generalist siblings. As showed by our study of individual efficiencies, these survival differences could neither be linked to an increased performance and activity of specialists, nor to those confounding factors (colony, individual size, and age at first foraging) included in our survival model. Similarly to honey bees^23^, precocious wasp foragers showed higher risk of death. Larger foragers tended to live longer. Moreover, coherently with the common assumption that foraging is a risky activity^23,27^, we found that wasps spending relatively more time in the field showed a reduced life expectancy. Yet, more specialized wasps did not expose themselves in the field more than the other foragers. Hence, the higher mortality risk experienced by specialist foragers could be explained by costs arising from individual specialization *per se*.

Diverse foraging tasks likely entail differential costs. Such costs might be levelled in controlled laboratory conditions but vary and play an important role in natural foraging conditions. We found that the collection of different resources required on average different times (Table S1). As suggested by the differences between years and similarities in the same foraging ground, the variable time costs for acquiring different resources are probably linked to their spatiotemporal distribution (SI). The transport of heavier loads increases energy expenditure and could accelerate ageing^49^. In yellowjackets, foraging for fluids (invariably the most common foraging task) means transporting the heaviest loads^32^. On the other hand, foraging for flesh implies other physical challenges. Typically, hunting yellowjackets pounce on their prey, a struggle ensues, and prey killing can be the culmination of an intense fight^34^. We also found that specialists could spend more time in the field for single foraging bouts. The length of a foraging trip varies according to the environmental conditions, and is the result of resource search, handling, and carrying times^46,50^. Possibly, the persistent search for a specific resource drives the specialized, fixated foragers to far, unknown, and riskier foraging grounds.

On the specialist-generalist continuum, animal groups’ assortment could be context-dependent. Colony composition may result from balancing selective forces. Within cooperative groups as in local populations, different phenotypes would be alternatively favoured^1,8,20^. As shown by our study system, specialization can be linked to a reduced efficiency and shorter life expectancy. Moreover, compared to generalists, specialists could be found to be less responsive, or less able to flexibly adjust their behaviour according to a perpetually changing environment and colony needs^51^. Yet, highly motivated specialists might be ultimately capable of discovering and acquiring valuable, and sometimes fundamental resources^7,45^. Rare behavioural phenotypes within insect colonies could be fundamental during unusual events and conditions^52^. Given the potential fitness advantages for a colony to include individual outliers, variation among individuals and exceptional phenotypes might be particularly favoured within eusocial species, even if selected traits are deleterious at the individual level.

## Supplementary information

Behaviourally specialized foragers are less efficient and live shorter lives than generalists in wasp colonies
