# Supplementary material for: Behaviourally specialized foragers are less efficient and live shorter lives than generalists in wasp colonies
Source: Sci Rep. 2019 Mar 29;9:5366. doi: 10.1038/s41598-019-41791-0 (PMC6441081; doi:10.1038/s41598-019-41791-0)
Supplement: Supplementary file 1 — Behaviourally specialized foragers are less efficient and live shorter lives than generalists in wasp colonies [file 41598_2019_41791_MOESM1_ESM.pdf]

Behaviourally specialized foragers are less efficient and live shorter lives than generalists in wasp colonies

Davide Santoro, Stephen Hartley, Philip J. Lester

Victoria University of Wellington, School of Biological Sciences, Wellington, New Zealand

Author for correspondence:
Davide Santoro
davide_santoro@tiscali.it

Supplementary information

Study sites and wasp foraging grounds. As far as it is known, most *Vespula* workers forage within 300 m from their nest [1,2]. We hence describe the sites surrounding the experimental colonies (see also Table S1) within this spatial range. In 2014, we studied colony A in the outskirts of a town, in the grounds of a Plant and Food Research Ltd. Laboratory in Lincoln, New Zealand's South Island. About 25% of the area was covered by buildings, parking lots and roads. The remaining area was vegetated and mostly covered by mown grass (70%), cultivated fields (20%) and several bush and tree patches (10%). A dozen of beehives were kept in the area. Most of the trees in the closest surroundings of colony A were *Quercus* spp. In 2017, we studied colonies B and C in a rural area by Pauatahanui village, New Zealand's North Island. The site was almost entirely vegetated, and the vegetation was more varied and structured than in Lincoln. Only one road and few buildings were present. About 40% of the area was covered by trees and bushes, and the rest by partially mown grass. A variety of fruit trees (e.g. *Pyrus*, *Malus*, *Prunus*, *Citrus* spp.), flowering *Eucalyptus* and *Salix* spp. were present within 50 m from the research facility, and one stream flowed few meters away from it.

1. Akre, R. D., Hill, W. B., Mac Donald, J. F. & Garnett, W. B. Foraging distances of *Vespula pensylvanica* workers (Hymenoptera: Vespidae). *J. Kansas Entomol. Soc.* **48,** 12–16 (1975).
2. Harper, G. A., Joice, N., Kelly, D., Toft, R. & Clapperton, B. K. Effective distances of wasp (*Vespula vulgaris*) poisoning using clustered bait stations in beech forest. *N. Z. J. Ecol.* **40,** 65–71 (2016).

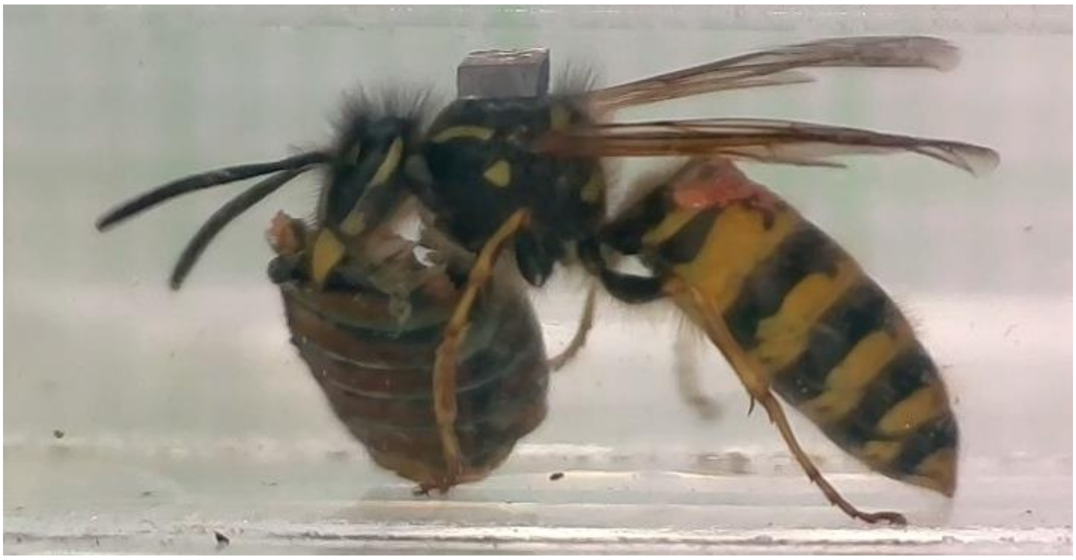

Fig. S1 *Vespula vulgaris* worker painted (on the abdomen) and provided with radio-frequency identification (RFID) tag (glued on the thoracic scutum). On the day of their emergence as adults, wasps were tagged, measured, and inserted in their colonies of origin. This individual is filmed while returning from a foraging trip, walking through the nest entrance and carrying a flesh load (insect abdomen) that will be fed to the carnivorous larvae. Snapshot image from video footage obtained by continuously filming the wasp nest entrance.

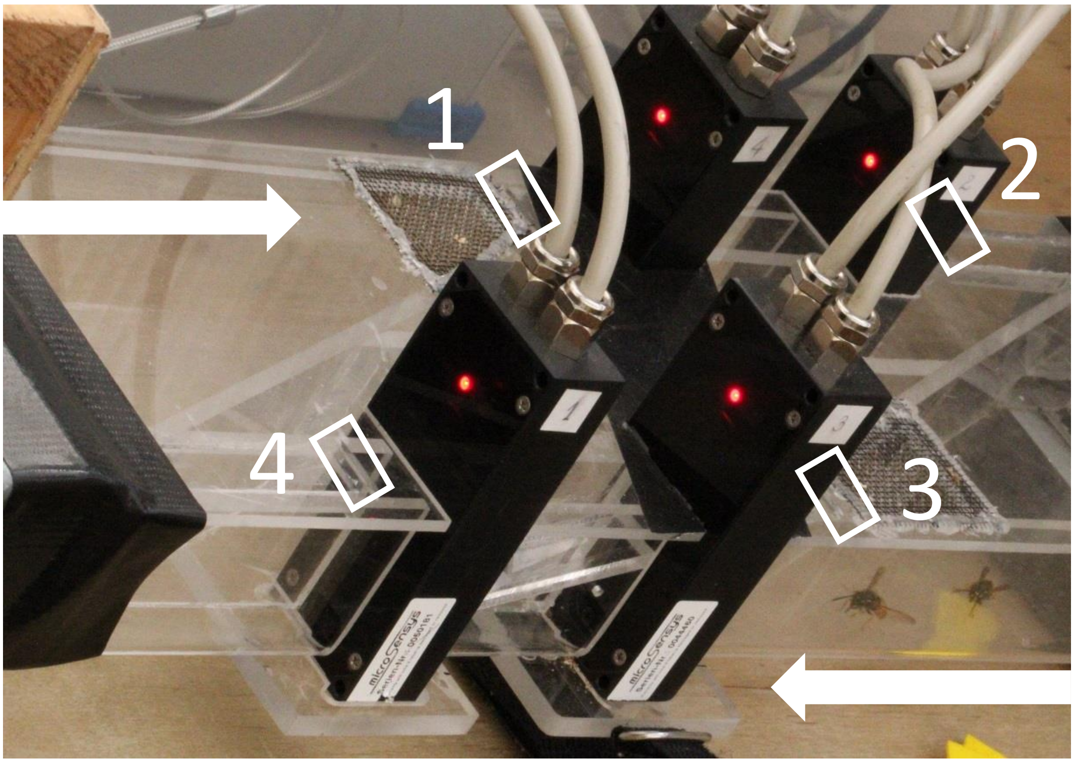

Fig. S2 Transparent entrance module attached to the *Vespula vulgaris* nest box (left), with separate lanes for wasps leaving and re-entering the nest (arrows). Each lane fitted two RFID scanners, recording the time and identity of the passing wasps provided with RFID tags. Four sliding gates could be inserted in slots on the module ceiling (numbered boxes) to block the wasps and isolate the scanners. Two returning foragers are visible (right). In 2017, when the nest boxes were closed and removed, the module was closed at its nest end, and gate #2 shut. The module was kept in place for the following week and worked as a trap for individuals returning to the nest after its removal.

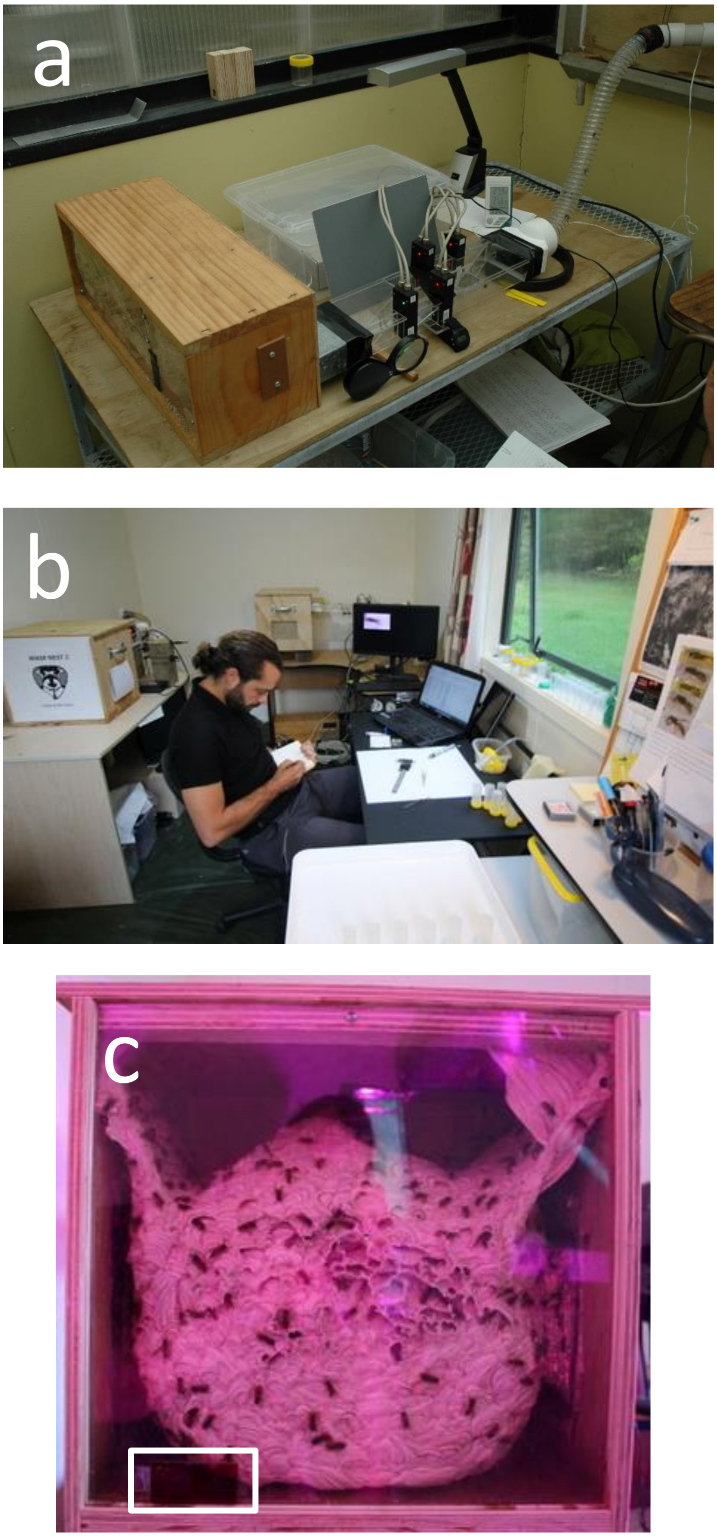

Fig. S3 *Vespula vulgaris* colonies set up in (a) 2014 (colony A) and (b) 2017 (colonies B and C). After being collected in the field, the nests were kept in boxes (c) in two different study sites (2014: Lincoln, 2017: Pauatahanui, New Zealand). The nest boxes were provided with a sliding door, windows with metal mesh screens, and an acrylic glass wall kept covered by a removable screen that allowed us to monitor the nests daily throughout data collection. This transparent wall had a little opening (5x5mm^2) closed by a door (c, bottom left box), through which the tagged individuals were inserted. Individually tagged wasps were free to forage in the field passing by a nest entrance provided with a RFID system (a) and a custom-made video camera (b).

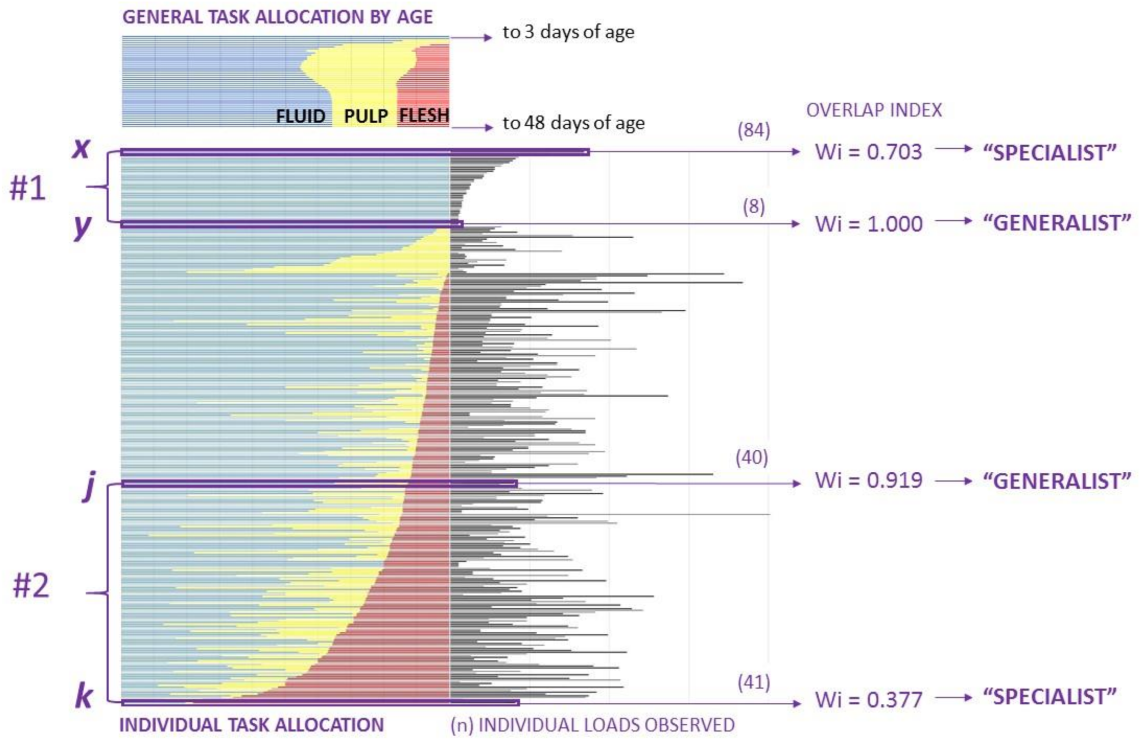

Fig. S4 *Vespula vulgaris* worker specialization measured with Petraitis' W_i index, quantifying the degree of overlap between individual and general task allocation by age (colony task allocation). W_i values range from 0 to 1. The lower the W_i value, the less overlap between the individual and the colony, hence, the more specialized the individual. The life-long individual task allocation was compared to the colony task allocation, considering the age when the individual performed the last trip. Average age-related variation in foraging task allocation is illustrated at the top left ("general task allocation by age") and corresponds to the age (temporal) polyethism. Visualization of observational data from colony A, where all the individually tagged wasps observed with at least 5 loads are represented (n individuals= 456; n loads= 15521).

Referring to the figure, as example (#1) consider two wasps (*x* & *y*) foraging only for fluids throughout their life, with *x* living and foraging much longer than *y*, dying three days old. If young individuals' loads were only fluids and the other foraging tasks were performed later in life, *x* would be a "specialist", but *y* a "generalist". On the other hand, (#2) consider one wasp (*j*) foraging for fluids 70% of the times. This foraging behaviour could be considered by itself "specialized". Yet, if we considered the task allocation at the colony level and knew that the vast majority of workers within the same colony were foraging for fluid with similar frequency, the individual would be a "generalist". Within the same colony, another individual (*k*) returning 70% of the times with flesh loads would be a "specialist" as this deviates further from the "average behaviour".

Table S1 The *Vespula vulgaris* colonies included in the study. For colony A, the RFID system was kept on for one week after the last RFID record. Colonies B and C were deep frozen when 12% of the workers provided with RFID tag were still alive (individuals recovered and censused in the survival analysis). Asterisks refer to those descriptive measures that would have been affected terminating the colonies by freezing (representing under-estimates). The estimates of lifespan and average number of trips over lifetime encompass only those wasps that performed at least one foraging trip (sortie longer than two minutes and shorter than eight hours). Measures are provided as mean ± standard deviation (minimum, maximum); median [first, third quartile].

**Common wasp colony**	**A**	**B**	**C**	**TOTAL**
**Year**	2014	2017	2017	
**Original nest location**	Christchurch	Stokes Valley	Upper Hutt	
**Original location coordinates**	(-43.611, 172.525)	(-41.183, 174.975)	(-41.105, 175.096)	
**Research facility location**	Lincoln	Pauatahanui	Pauatahanui	
**Research location coordinates**	(-43.640, 172.475)	(-41.089, 174.917)	(-41.089, 174.917)	
**Study period**	20/01/2014-17/03/2014	10/2/2017-20/03/2017	28/2/2017-28/03/2017	
**RFID tagging period**	20/01/2014-31/01/2014	10/2/2017-3/3/2017	28/2/2017 and 14/3/2017	
**N individually tagged workers (N RFID tags)**	630 (300)	666 (666)	361 (361)	**1657**
**N tagged workers recovered at the end of the study**	0	70*	54*	**124**
**Hours of observation**	145	180	140	**465**
**N loads observed**	25926	50530	25344	**101800**
**N loads carried by individually tagged workers**	15521	17574	6537	**39632**
**N loads carried by non tagged workers in 10 min intervals**	10405	32956	18807	**62168**
**N foraging trips (2min-8h) recorded by RFID system**	32242	22382	9135	**63759**
**Trip length for liquid (minutes)**	27 ± 24 ; 22 [12, 32]	19 ± 26 ; 13 [6, 22]	16 ± 21 ; 11 [5, 18]	
**Trip length for pulp (minutes)**	22 ± 18 ; 18 [10, 29]	25 ± 37 ; 15 [8, 25]	23 ± 33 ; 13 [7, 23]	
**Trip length for flesh (minutes)**	21 ± 19 ; 15 [8, 28]	28 ± 40 ; 17 [10, 28]	27 ± 42 ; 14 [8, 24]	
**RFID foragers' average lifespan (days)**	30 ± 8 (14, 50); 31 [25, 35]	17 ± 6 (7, 37); 16 [13, 19]*	15 ± 3 (6,28); 15 [13, 17]*	
**RFID foragers' average number of trips over lifetime**	171 ± 164 (1, 805); 204 [122, 299]	37 ± 75 (1, 614); 17 [7, 37]*	29 ± 62 (1, 583); 20 [7, 45]*	
